# Requesting physicians' experiences regarding infectious disease consultations

**DOI:** 10.1186/1471-2334-11-62

**Published:** 2011-03-14

**Authors:** Patricia Pavese, Elodie Sellier, Laurent Laborde, Stéphane Gennai, Jean-Paul Stahl, Patrice François

**Affiliations:** 1 Infectious Diseases Unit, Grenoble University Hospital, F-38043, Grenoble, France; 2 Quality of Care Unit, Grenoble University Hospital, F-38043, Grenoble, France; 3 UJF-Grenoble 1/CNRS/TIMC-IMAG UMR 5525, Grenoble, F-38041, France

## Abstract

**Background:**

Solicited consultations constitute a substantial workload for infectious disease (ID) specialists in the hospital setting. The objectives of this survey were to describe requesting physicians' experiences regarding ID consultations.

**Methods:**

A cross-sectional survey was conducted in a university-affiliated hospital in France in 2009. All physicians were eligible (*n *= 530) and received a self-administered questionnaire. The main outcomes were reasons for request and opinion. Secondary outcomes were frequency of request and declared adherence to recommendations.

**Results:**

The participation rate was 44.7% (237/530). Among the responders, 187 (79%) had solicited the ID consultation service within the previous year. Ninety-three percent of the responders (173/187) were satisfied with the ID consultation. The main reasons for requesting consultations were the need for therapeutic advice (93%), quality of care improvement (73%) and the rapidity of access (61%). ID consultations were requested several times a month by 52% (72/138) of senior physicians and by 73% (36/49) of residents (*p *= 0.01). Self-reported adherence to diagnostic and therapeutic recommendations was 83% and 79%, respectively.

**Conclusion:**

The respondent requesting physicians expressed great satisfaction regarding ID consultations that they requested principally to improve patient care and to assist in medical decision making.

## Background

In order to improve the quality of care, recommendations given by specialists to physicians of different specialties, or from specialists to general practitioners, are an important part of medical practice. The positive impact of such practice on the quality of care and colleague teaching has been studied for various specialties including infectious diseases (ID) [1-4].

The growing problem of nosocomial infections [5] and micro-organism resistance to antibiotics [6] has enhanced the role of ID specialists in hospitals. Their positive impact on patient care and infection control has been demonstrated [1,7,8]. Moreover, ID specialists can improve the effectiveness of care by recommending a more appropriate use of antibiotics [9,10], and, ultimately, shorten the median length of stay and improve patient outcomes [3,7].

Solicited consultations are part of the everyday practice and a substantial part of ID specialists' workload in hospitals [2,11]. The most frequent purposes of requests are related to therapeutic and diagnostic recommendations. Despite the frequency of these solicited consultations, little is known about physicians' needs, attitudes, and opinions toward these consultations.

This cross-sectional survey was designed to describe requesting physicians' experiences regarding the ID consultation. This included the frequency and reasons for requests, opinions, adherence to recommendations and suggestions for improving the ID consultation.

## Methods

### Study design

We carried out a cross-sectional survey among the physicians of a 2200-bed university-affiliated hospital in France, between March 2009 and May 2009. The Institutional Review Board (Comité d'éthique des centres d'investigation clinique de l'inter-région Rhône-Alpes-Auvergne. Centre de Grenoble; IRB 5921) waived the requirement for informed consent and approved the study protocol.

### Setting

ID consultations were provided by a dedicated team, working within a specialized unit providing tropical disease consultation, vaccination for travelers and ID consultation. The consultation service was available 7 days a week, 24 h a day, through a hotline via a dedicated cellular phone. It could also be reached via the secretariat, e-mail and fax. Working hour coverage was provided by both a resident and a board-certified specialist, whereas overnight and weekend coverage was only provided by one of the five board-certified specialists working in the ID department. The specialist specified his recommendations either informally (without examining the patient) or formally (examining the patient before giving recommendations). Recommendations given by the ID resident were always supervised by a board-certified ID specialist. Although solicited consultations were routinely provided to community primary-care physicians, we focused on physicians working in the hospital for this study.

### Population

We enrolled all physicians, residents, and senior physicians working in a clinical ward in the Grenoble University Hospital (France) and registered on the physician working list on January 1^st^, 2009. We excluded all physicians working in a non-care-providing unit such as radiology, laboratory, and public health units.

### Data collection

All physicians were sent a self-administered questionnaire via their professional e-mail address. Physicians who did not respond to the survey received up to five e-mail reminders before being considered a nonresponder. The questionnaire included questions regarding demographic characteristics (age, sex, working status, department, specialty), the frequency of ID consultation requests (never, less than once a year, between once a month and once a year, between once a week and once a month and more than once a week), the route and reasons for requesting consultations, their adherence to recommendations and their opinion on the consultations. In addition, an open-ended question was asked at the end of the questionnaire where physicians could add suggestions for improving ID consultations. Demographic characteristics were checked for physicians who did not respond to the survey using the hospital database.

### Study Outcomes

Our primary study outcomes were the physicians' reasons for requests and their opinion on the consultations. We asked responders to indicate their agreement with nine potential reasons for requesting consultations. Their opinion on the consultations was assessed using a four-point scale for five items that were related to the pertinence of diagnosis and therapeutic recommendations, the atmosphere during the exchanges, and overall satisfaction. Physicians were asked whether they would request future consultations and whether they would recommend that their colleagues ask for ID consultations. The secondary outcomes were frequency of consultation requests and declared adherence to therapeutic and diagnostic recommendations.

### Statistical analysis

Descriptive statistics were presented as numbers and percentages for categorical variables or median and 25^th ^and 75^th ^percentiles for continuous variables. Differences in characteristics were compared using the χ^2 ^or Fisher exact tests where appropriate for categorical variables and the Wilcoxon rank sum test for continuous variables.

We compared baseline characteristics between responders and nonresponders as well as between nonresponders and responders and users of ID consultations.

Two-sided *p*-values of less than 0.05 were considered statistically significant. Analyses were performed using Stata statistical software (version 10.0, Stata Corp, College Station, TX, USA).

## Results

Five hundred thirty professionals were eligible, including 383 senior physicians and 147 residents. A total of 237 professionals (45%) participated in the study. There were no differences in baseline characteristics between responders and nonresponders except for hospital department (Table 1). Responders were more likely to work in medical departments than nonresponders.

**Table 1:** Characteristics of physicians who did not respond to the survey, who responded to the survey, and who used the infectious disease consultation.

Variable	Nonresponders (A) (*n *= 293)		Responders (B) (*n *= 237)		Responders and users (C) (*n *= 187)		*P-value*	
							A vs. B	A vs. C
Age, y, median (IQR)	39	(29-50)	37	(29-47)	37	(29-47)	0.44	0.24
Women, *n *(%)	158	(53.9)	123	(51.9)	99	(52.9)	0.64	0.83
Working status, *n *(%)								
Senior physician	207	(70.6)	176	(74.3)	138	(73.8)	0.36	0.45
Resident	86	(29.4)	61	(25.7)	49	(26.2)		
Department, *n *(%)								
Medicine*	121	(41.3)	125	(52.7)	98	(52.4)	0.03	0.04
Surgery	93	(31.7)	63	(20.7)	54	(28.9)		
ICU	79	(27.0)	49	(20.7)	35	(18.7)		

Abbreviations: IQR, interquartile range; ICU, intensive care unit

* Rehabilitation or long-term care units were categorized as medical departments.

Among the responders, 187 (79%) had requested at least one ID consultation within the previous year. They reached the ID specialist preferably by calling on the cell phone (89.8%) or calling the secretariat (5.7%) or by writing an e-mail (4.5%). The most frequent reasons for requesting consultations were to obtain therapeutic recommendations (93%) and to improve the quality of care (73%) (Table 2). Rapidity of access, diagnostic recommendations, and updating knowledge were reported by about six out of ten physicians. There were no statistical differences between residents and senior physicians regarding the reasons for consultation. Sharing stress related to care was the least reported reason (17%).

**Table 2:** Reasons for requesting infectious disease consultations among 187 responders and users.

	Senior physicians *n *= 138		Residents *n *= 49	
	
Potential reason	***n***^**a**^	%	***n***^**a**^	%
Therapeutic recommendation	128	(92.8)	46	(93.9)
Quality of care improvement	101	(73.2)	36	(73.5)
Rapidity of access	84	(60.9)	30	(61.2)
Diagnostic recommendation	83	(60.1)	30	(61.2)
Update knowledge	80	(58.0)	31	(63.3)
Preventive recommendation	50	(36.2)	15	(30.6)
Share responsibility of care	43	(31.2)	15	(30.6)
Transfer a patient to infectious care unit	29	(21.0)	9	(18.4)
Share stress related to care	24	(17.4)	8	(16.3)

^a ^*n *represents the number of physicians who agreed with the prespecified reason

Among the responders and users, ten did not indicate the frequency of ID consultation solicitations. Sixty-one percent of the others (108/177) declared that they requested consultations more than once a month and 11% more than once a week (19/177). Consultations were requested several times a month by 52% (72/138) of senior physicians and by 73% (36/49) of residents (*p *= 0.01). Respondent physicians working in surgical or medical units were also more likely to request a consultation more than once a month [71% (37/52) and 68% (63/92), respectively] than those working in intensive care units [24% (8/33)] (*p *< 0.001). The difference between physicians in surgical and medical units was not significant (*p *= 0.44). Full adherence with therapeutic or diagnostic recommendations was declared by 83% and 79% of physicians, respectively, with no difference between residents and senior physicians (Table 3).

**Table 3:** Adherence and satisfaction regarding infectious disease consultation.

	Senior physicians *n *= 138		Residents *n *= 49		*p*-value
	
Variables	*n*	**%**^**a**^	*n*	**%**^**a**^	
**Declared adherence to recommendations**					
Therapeutic recommendations					0.89
I fully followed the recommendations	113	81.9	42	85.7	
I partially followed the recommendations	17	12.3	4	8.2	
I rarely followed the recommendations	0	-	0	-	
Diagnostic recommendations					0.12
I fully followed the recommendations	112	81.2	35	71.4	
I partially followed the recommendations	16	11.6	10	20.4	
I rarely followed the recommendations	0	-	0	-	
**Opinion**					
Pertinence of recommendations					0.15
Very good	80	58.0	32	65.3	
Good	48	34.8	13	26.5	
Poor	0	-	1	2.0	
Very poor	0	-	0	-	
Atmosphere during exchanges					0.94
Very good	91	65.9	32	65.3	
Good	38	27.5	13	26.5	
Poor	0	-	0	-	
Very poor	0	-	0	-	
Overall satisfaction					0.17
Very satisfied	80	58.0	31	63.3	
Somewhat satisfied	49	35.5	13	26.5	
Somewhat dissatisfied	0	-	1	2.0	
Very dissatisfied	0	-	0	-	
Expect to request consultation again					0.68
Certainly	122	88.4	44	89.8	
Probably	7	5.1	1	2.0	
Probably not	0	-	0	-	
Certainly not	0	-	0	-	
Recommendation to a colleague					0.43
Certainly	123	89.1	42	85.7	
Probably	5	3.6	3	6.1	
Probably not	0	-	0	-	
Certainly not	0	-	0	-	

^a ^The percentages for each item did not total 100% because several physicians did not answer all items

Physicians' opinions were very positive. Fifty-nine percent of physicians (111/187) were very satisfied and 33% (62/187) somewhat satisfied with the consultations. Positive ratings reached 93% of responders concerning the pertinence of recommendations, the atmosphere during the exchanges, the intention of requesting another ID consultation, and the recommendation of the consultation service to a colleague (Table 3). There was only one negative opinion concerning overall satisfaction and the pertinence of recommendations.

Twenty-six physicians (22 senior physicians and four residents) answered the open-ended question "Do you have any suggestions to improve the infectious disease consultation?" allowing us to identify 27 items (Table 4). Thirteen answers were true suggestions, whereas 14 were either positive (*n *= 8) or negative (*n *= 6) comments. The most frequent suggestions were to provide direct access to a board-certified ID specialist and to improve the traceability of specialists' recommendations. Positive comments were mostly related to the quality and usefulness of the ID consultation.

**Table 4:** Requesting physicians' free comments to the survey.

Item	Frequency (n)
Suggestions	
Recommendations should be given directly by the board-certified infectious disease specialist	7
The traceability of recommendations should be improved	3
Other specialists should develop hotline consultations	1
Teaching sessions should be performed on frequently asked questions	1
Guidelines should be published on the hospital's computer network	1
Positive comments	
Infectious disease consultation perfect, practical, or essential	7
Infectious disease specialist can hospitalize the patient in the infectious disease care unit if necessary	1
Negative comments	
The effort in self-training is reduced by facility of access	2
Recommendations are excessively standardized given that the patient is not examined	2
Computerized guidelines are not used	1
There are contradictions between board-certified infectious disease specialist and resident recommendations	1

## Discussion

In this study, 61% of physicians declared that they requested an ID consultation more than once a month. They were physicians working in medical, surgical, and intensive care units, although those working in medical and surgical units declared requesting more consultations than the others. This is concordant with a previous study conducted in the same setting showing that 55% of the 2933 consultations requested of ID specialists over 1 year came from medicine or rehabilitation units, 41% from surgical units and 2% from intensive care units [12]. We could have expected a majority of requests from surgeons as they are less experienced with IDs. However, surgeons were less numerous than physicians working in medical units. Furthermore, physicians working in medical units in French university hospitals are often highly specialized. Although they are used to managing common IDs themselves, most hospitalized patients have complex illnesses that ID specialists are specifically trained to assess [1,13].

The majority of responders (93%) was satisfied with ID consultations and had a positive opinion on the pertinence of recommendations. This is much higher than a previous study that showed only 53% satisfaction for curbside consultations among primary care physicians and medical subspecialists [14]. The consultation with an easily reached specialist seems to be an appropriate answer to an important need for the hospital community given the frequency of ID encountered among inpatients [13,15]. Consultations were provided by a dedicated team used to informal and formal consultations for inpatients in the different departments. It can be assumed that this team had developed the basic principles of good communication with the requesting physicians, which has been shown to increase adherence with recommendations [16,17].

The most frequent reasons for requesting ID specialist consultations were to obtain therapeutic advice and to improve quality of care, consistent with other studies [12,13]. Another frequent reason, declared by nearly two-thirds of physicians, was the rapidity of access to the consulting ID specialist. Although the rapidity of access by itself may not be the only reason for requesting a consultation, it can obviously facilitate it. Indeed, if an ID specialist can be reached easily and gives an appropriate recommendation, it is likely that non-ID specialists will contact him rather than seeking other sources of information, which is more time-consuming. This result is highlighted by the predominance of reaching specialists via the hotline and much less often via other routes. The importance of communicating directly at the time a consultation is requested has been reported previously [18].

In the present study, self-reported adherence to therapeutic and diagnostic recommendations was 83% and 79%, respectively. These figures are close to those found in the Sellier *et al*. study conducted in the same hospital where adherence to ID recommendations was objectively assessed by an independent physician and was measured at 88% for therapeutic recommendations and 72% for diagnostic recommendations [7]. In the free comments, several physicians found that recommendations were sometimes overly standardized because the specialist did not examine the patient. Though they found the consultations useful, they decided not to follow the recommendations precisely because they believed they knew the patient better. Informal consultations make up 46% of ID consultations dispensed in this setting [12]. ID specialists seem to provide informal consultations for less complex cases in comparison with formal consultations [19]. It is likely that the most important recommendations would not change with additional clinical information and that standard recommendation may be justified. Moreover, a previous study showed that adherence to ID specialist recommendations and patient outcomes were comparable between formal and informal consultations [19]. It should be noted that informal consultations seem to require more experience on the part of physicians, as it has been shown that ID residents tended to provide informal consultations less than board-certified specialists.

Another frequent suggestion made by the requesting physicians was to reach only board-certified ID specialists and not residents. Coverage during working hours by both a resident and a board-certified specialist was a choice made in order to reduce the time to obtaining the answer. The substantial workload of this ID consultation requires an operational mode based on ease and rapidity of access. Furthermore, it has been demonstrated that ID trainees provide valuable recommendations during consultations [20]. Three requesting physicians expressed a concern about the traceability of recommendations given by telephone. Shared access to the patient's computerized medical chart can be a way to decrease the risk of liability. Also, in an informal consultation, it could be suggested that the ID specialist write a note in the chart mentioning the reason why the patient did not need to be examined.

The majority of physicians declared using the consultation service to update knowledge. Although the educational impact of specialist consultation has often been described [1,21,22], it is worthwhile to consider another aspect underlined in the present study. The rapidity and the guaranteed relevance of the recommendations made by the ID specialist may incite the requesting physician to rely on the consultation and not seek the answer to his request on his own.

Our study has several limitations. Forty-five percent of the physicians having received the survey responded to it. It is likely that those who participated in the study were the most likely to be aware of this consultation service, but we cannot exclude that physicians who answered may have had a better opinion regarding ID consultations than those who did not. Thus, satisfaction might have been overestimated in this study. By comparing the demographic characteristics of responders and nonresponders, we found certain differences between them. They may consequently represent two different populations. Moreover, although few responders answered the open question, the comments made should help specialists of the team to consider how they could improve their practice. Finally, our survey was carried out in one university hospital in France, and we cannot exclude that the findings may be different in other settings and geographic locations.

## Conclusions

The respondent requesting physicians expressed great satisfaction regarding the ID consultation that they asked principally for improving patient care and medical decision making. The ID consultation responds to the needs of the medical community and the hotline, although perfectible, appears to be a good way to satisfy these requests.

## Competing interests

The authors declare that they have no competing interests.

## Authors' contributions

PP, JPS and PF participated in the conception and design of the study. PP, JPS and SG contributed to the acquisition of data. LL, ES, PP and PF contributed to analysis and interpretation of the data. LL, PF and ES were involved in drafting the manuscript. All authors read and approved the final manuscript.

## Pre-publication history

The pre-publication history for this paper can be accessed here:

http://www.biomedcentral.com/1471-2334/11/62/prepub
